# Midwives Registration Association

**Published:** 1894-01-20

**Authors:** Robert Boxall, Rowland Humphreys

**Affiliations:** 29, Weymouth Street, W. Hon. Sec.; 27, Fellows Road, N.W. Hon. Sec.


### EDITOR'S LETTER-BOX.

[Our correspondents are reminded that proxility is a great bar to publication, and that brevity of style and conciseness of statement greatly facilitate early insertion.]

### MIDWIVES' REGISTRATION ASSOCIATION.

Dear Sir,—We beg to call your attention to the above Association, which has been formed in order to obtain a practical solution of what is known as the "Midwives' Question." The want of a society specially devoted to this object has been much felt all through the agitation wh'ch has been going on intermittently for more than twenty years. The formation of this association has, therefore, been welcomed by those who rightly appreciate the importance of the question.

Since two Select Committees of the House of Commons have recently reported in favour of an Act to render compulsory the registration of midwives, such an association has been still more needed in order that medical practitioners may be recognised as the proper persons to undertake the duties of examining midwives, and of supervising them in their practice.

Our association, therefore, solicits the co-operation of all those members of the profession who agree that legislation is required, and who think, if desirable, that the medical profession should have a voice in the matter.

An Executive Committee, consisting of both general practitioners and consultants, has been appointed, and is at present engaged in considering questions raised by the prospect of legislation. Medical societies interested in the matter are represented on this committee by duly-appointed delegates.

We shall feel greatly obliged to medical practitioners in any part of the country who will furnish us with local information bearing on the question.

We append copies of papers now in circulation.—We are, dear Sir, yours truly,

Robert Boxall, M.D., 29, Weymouth Street, W.

Rowland Humphreys, 27, Fellows Road, N.W.

